# The contribution of drug import to the cost of tuberculosis treatment: A cost analysis of longer, shorter, and short drug regimens for Karakalpakstan, Uzbekistan

**DOI:** 10.1371/journal.pgph.0000567

**Published:** 2022-08-03

**Authors:** Stefan Kohler, Norman Sitali, Jay Achar, Nicolas Paul

**Affiliations:** 1 Institute of Social Medicine, Epidemiology and Health Economics, Charité –Universitätsmedizin Berlin, Corporate Member of Freie Universität Berlin and Humboldt-Universität zu Berlin, Berlin, Germany; 2 Heidelberg Institute of Global Health, Faculty of Medicine and University Hospital, Heidelberg University, Heidelberg, Germany; 3 Médecins Sans Frontières, Berlin, Germany; 4 Department of Global Public Health, Karolinska Institutet, Stockholm, Sweden; University College London, UNITED KINGDOM

## Abstract

Tuberculosis (TB) programs depend on a continuous supply of large amounts of high-quality TB drugs. When TB programs procure TB drugs from international suppliers, such as the Global Drug Facility, they can incur import costs for international transport, customs clearance, and national transport. We assessed the drug costs and import costs of 18 longer (≥18 months), 10 shorter (9–12 months), and 8 short (≤6 months) drug regimens for drug-sensitive (DS) and multidrug-resistant (MDR)-TB treatment. Costs per regimen were estimated by multiplying recommended drug amounts with 2021 Global Drug Facility prices and drug import costs of a TB program in Karakalpakstan, Uzbekistan. The standard short-course treatment of DS-TB requires taking 730 fixed-dose combination tablets, which weigh 0.79 kg and cause an import cost of $4.19 (9.8% of the regimen’s drug cost of $43). A new 4-month DS-TB regimen requires taking 1358 tablets, which weigh 1.1 kg and cause an import cost of $6.07 (2.6% of the regimen’s drug cost of $233). MDR-TB regimens that last between 24 weeks and 20 months involve 546–9368 tablets and injections. The drugs for these MDR-TB regimens were estimated to weigh 0.42–96 kg and cause an import cost of $2.26–507 per drug regimen (0.29–11% of a regimen’s drug cost of $360–15,028). In a multivariable regression analysis, an additional treatment month increased the import cost of a drug regimen by $5.45 (95% CI: 1.65 to 9.26). Use of an injectable antibiotic in a regimen increased the import cost by $133 (95% CI: 47 to 219). The variable and potentially sizable import costs of TB regimens can affect the financial needs of TB programs. Drug regimens that are shorter and all-oral tend to reduce import costs compared to longer regimens and regimens including an injectable drug.

## 1 Introduction

In 2020, 1.3 million deaths worldwide were attributed to tuberculosis (TB), and drug-resistant TB continued to be considered a public health threat [1]. Compared to the standard 6-month treatment of drug-susceptible (DS)-TB [2], the treatment of multidrug-resistant (MDR)-TB can last up to two years, involve injectable antibiotics, and include more toxic antibiotics [3–5]. Conventional MDR-TB treatment lasted ≥18 months and required that tablets and injections are taken [3]. It has been associated with adverse side effects, particularly ototoxicity, frequent treatment discontinuation, and low treatment success rates [6–8]. Newer MDR-TB treatments are based on shorter 9–12-month regimens with an injectable antibiotic, recommended by the World Health Organization (WHO) for certain people with MDR-TB in 2016 [3], and longer all-oral regimens, recommended by the WHO over conventional MDR-TB regimens in 2019 [4]. With regimens that include new and re-purposed TB drugs, all-oral MDR-TB treatment in 6–12 months meanwhile also appears feasible in many instances. The WHO recommended 9–12 months regimens with bedaquiline instead of injectables in 2020 [5], and it endorsed the programmatic use of 6-month all-oral MDR-TB regimens in May 2022 [9] based on results from recent clinical trials [10–13]. People with DS-TB may be treated with a new 4-month regimen treatment since June 2021 [14–17]. Additional MDR-TB treatments in 6 months or less time are and have been evaluated in clinical trials (e.g., [18–22]).

To be able to administer different TB regimens at the point of care, TB programs depend on a continuous supply of high-quality TB drugs [23]. Related to limited domestic procurement options or a risk of supply with substandard drugs [24], TB programs commonly rely on the purchase and import of TB drugs from few manufacturers that are quality-assured by the WHO’s prequalification program or a national medicines regulatory authority [25–27]. Often, quality-assured TB drugs are supplied through the Global Drug Facility at a fixed and subsidized price that is exclusive of delivery costs [28, 29]. When TB programs pay for international and national transport or for customs-related charges (e.g., customs, temporary storage during customs clearance or a customs broker) during their procurement process, they incur import costs. The import costs for TB drugs and other medical supplies can be sizable [30], particularly when importing to low-income and landlocked countries [31–33], but have been rarely studied in detail and for specific health programs [34–41].

TB regimen costs have been studied before, but previous assessments have calculated regimen costs without considering drug import costs [42–48]. This study extends previous assessments of DS-TB and MDR-TB regimen costs by estimating the costs of drug regimens including regimen-specific import costs. We build on our previous micro-costing study, in which we estimated drug-specific import costs to a TB program in Karakalpakstan, Uzbekistan [39]. Our aims were, first, to estimate the number of tablets and injections required for different TB regimens; second, to estimate regimen costs based on 2021 TB drug prices and, third, to estimate regimen import costs for a TB program in Karakalpakstan, Uzbekistan.

## 2 Methods

### 2.1 Study design

We performed a cost analysis of TB drug regimens for adults from the perspective of a TB program in Karakalpakstan, Uzbekistan. The drug costs and drug import costs of 3 DS-TB and 36 MDR-TB regimens were calculated in a bottom-up costing approach. To predict the costs of a drug regimen, all TB drugs required by the regimen were multiplied with drug unit prices and drug unit import costs. Placebo tablets from TB trial regimens were excluded. Costs were analyzed for TB drug regimens that are or have been used in the TB program in Karakalpakstan and for additional TB drug regimens, including drug regimens which may be used in other TB programs and from 6 recent TB trials (Nix-TB trial [11], NExT Study [18], TBTC Study 31 [17], TB-PRACTECAL [12], ZeNix trial [13], and BEAT TB trial [19]).

### 2.2 Study setting

Uzbekistan is among the high MDR-TB burden countries identified by the WHO [1]. In 2018, 23,000 people newly developed TB. Of those, 6700 (29%) were diagnosed with drug-resistant TB. About 1.9 million people of Uzbekistan’s 33 million population live in the northwestern Republic of Karakalpakstan [49, 50]. Médecins Sans Frontières (MSF) has been supporting TB care in Karakalpakstan since 1998. The TB program in Karakalpakstan is an active research site and has been previously described (see, e.g., [51, 52]). In addition to conventional DS-TB and MDR-TB regimens, shorter MDR-TB regimens are offered and evaluated. About 40% of the people treated for TB in the program in 2015 had a weight of 55–70 kg.

Drugs and other medical supplies for the TB program in Karakalpakstan are regularly shipped from an MSF procurement unit in Amsterdam to Karakalpakstan as humanitarian goods [53]. Items are flown from a central storage facility in Amsterdam to Tashkent. At Tashkent airport, items are kept in storage until customs clearance is completed. There are no customs charges, but interim cargo storage and the use of a customs broker cause customs-related costs. After customs clearance, the imported medical supplies are transported in a rented truck to a storage facility in the Karakalpak capital Nukus [39].

### 2.3 Data sources

TB regimens and dosing of required TB drugs were extracted from TB program guidelines for Karakalpakstan [54] and Uzbekistan [55], WHO guidelines [5], TB trial registrations on ClinicalTrials.gov [11–13, 17–19], and MSF Access Campaign reporting. All-oral TB regimens were extracted from the MSF Access Campaign report 2020, TB regimens including an injectable antibiotic were extracted from MSF Access Campaign reports 2018–20 [42–44]. TB drug prices and drug formulations were extracted from the October 2021 Global Drug Facility Medicines Catalog (e.g., $21.65–27.70 for 672 film-coated tablets of ethambutol 400 mg packaged in a blister) [56]. When the Global Drug Facility reported a price range, the lowest price available to any country, including free goods and other discounts, was chosen. As the latest Global Drug Facility Medicines Catalogs no longer contain the injectable antibiotics capreomycin and kanamycin, their prices were extracted from the August 2018 Global Drug Facility Medicines Catalogs [57]. August 2018 drug prices were adjusted for 2018–2021 price changes in advanced economies using the gross domestic product deflators from the World Economic Outlook Database of the International Monetary Fund [58].

Unit import costs of TB drugs stem from a previous micro-costing study of the import costs to the TB program in Karakalpakstan [39]. In this micro-costing study, we first collected all freight charges and costs related to customs clearance for a major shipment to the TB program in 2016. Secondly, we allocated the total import cost of this shipment to all medical supplies it contained. To allocate the total import cost to individual items, we used their unit weights and the quantities imported within the same item line. Unit weights were extracted from an MSF list of frequently used and ordered medical items, called the MSF Green List [59]. Drug unit weights from the MSF Green List included a share of the primary item packaging (e.g., a plastic jar or a blister) but no share of the secondary packaging used for shipping. Based on our prior micro-costing results, we multiplied the unit weight of TB drugs imported to the TB program in Karakalpakstan with an estimated gross-to-net weight ratio of 1.11 to account for secondary packaging required during the shipment process of medical standard cargo (**Table A in S1 File**).

For the study at hand, we converted our prior import cost estimates from 2016 Euro (€) to 2021 US Dollar ($) using exchange rates from the International Financial Statistics and gross domestic product deflators from the World Economic Outlook Database of the International Monetary Fund [58, 60]. Land freight and customs-related costs were converted from Euro in Uzbek Soms (UZS) based on the 2016 exchange rate, before adjusting for 2016–2021 price changes in Uzbekistan. Air freight costs were adjusted for 2016–2021 price changes in advanced economies. Currencies were then converted to US Dollar using 2021 annual exchange rates: $1 = €0.845 = UZS10609.

### 2.4 Data analysis

To estimate the weight of all drugs imported for a TB regimen, we multiplied the required drugs units (e.g., 243 HRZE and 487 HR fixed-dose combination tablets) with their respective unit weights (e.g., 1.2 g and 1.0 g) and then summed these weights up by regimen. To estimate the drug cost and import cost of a TB regimen, we multiplied the number of drug units (e.g., 1 tablet of isoniazid 300 mg) that are required for one treatment course with unit prices (e.g., ¢1.8 per tablet) and unit import costs (e.g., ¢0.60 per tablet), respectively, and then summed up these costs by regimen. Univariable and multivariable linear regression models with Huber-White robust standard errors were used to assess the relationship between the import and drug costs and the characteristics of a TB regimen (i.e., number of tablets and injections in a regimen, regimen duration, and all-oral regimen or regimen including an injectable antibiotic). Wald tests were used to assess the equality of regression coefficients. Confidence intervals for nonlinear combinations of coefficients used to estimate when TB regimens cost the same were based on the delta method.

Import costs were imputed for some TB drugs that were part of the assessed TB regimens but not part of the previously studied shipment. Transport costs were imputed by multiplying the drug unit weight with the average transport costs of standard cargo. Missing customs-related costs were replaced by the median customs-related cost of the TB drugs in the previously studied shipment of medical supplies to the TB program [39]. Unit weights not available from the MSF Green List were imputed by multiplying the dose of the active ingredient with the median weight-per-dose of similar items (**Table B in S1 File**). All analyses were performed in Stata 15.1 SE.

### 2.5 Costing assumptions

We assumed shortest TB regimen durations and that TB drugs are procured at the lowest Global Drug Facility prices. Drugs to treat side effects were not considered. Drug import costs were derived in a prior costing study whose cost allocation assumptions have been previously described [39]. The number of tablets and injections in a TB regimen were calculated based on recommended dosing and available drug formulation for an adult weighing about 60 kg. Where drug regimens were formulated in weeks rather than months, we converted treatment durations based on an average month with 30.4167 days.

### 2.6 Ethical considerations

No ethical approval was sought as this cost analysis used secondary data.

## 3 Results

### 3.1 Unit weights, unit prices and unit import costs of TB drugs

Unit weights (0.2–38 g), unit prices (¢1.8–$2.94), unit import costs (¢0.12–20), and percentage unit import costs (0.12–34% of the unit price) varied across TB drugs. The highest unit weights (38 g, 36 g, and 34 g) and therewith related the highest unit import costs (¢20, ¢19, and ¢18) were associated with the injectable antibiotics capreomycin 1 g, imipenem/cilastatin 500 mg/500 mg, and kanamycin 1 g that are contained in vials. The lowest unit weights (0.22 g, 0.33 g, and 0.58 g) and lowest unit import costs (¢0.12, ¢0.18, and ¢0.31) were associated with the oral antibiotics clofazimine 100 mg, bedaquiline 100 mg, and rifapentine 150 mg. The TB drugs isoniazid 300 mg, pyrazinamide 500 mg, and levofloxacin 500 mg had the highest unit import costs relative to their unit price (34%, 33%, and 26% of the unit price of ¢1.8, ¢2.0, and ¢4.5, respectively; **Table 1**).

**Table 1:** Unit prices and unit import costs of tuberculosis (TB) drugs for a TB program in Karakalpakstan, Uzbekistan.

TB drug and formulation	Abbreviation	Import weight (g)	Unit price (¢)	Unit import cost (¢)	Import cost (% of price)	Adult dose per day
**Single medicines**						
Amikacin 500 mg/2 ml	**Am**	12	59	6.5	11	2
Amoxicillin/clavulanic acid 500 mg/125 mg	Amx/Clv	2.7	9.3	1.4	15	2
Bedaquiline 100 mg	Bdq	0.33	145	0.18	0.12	0–4
Capreomycin 1 g	**Cm**	38	230	20	8.7	1
Clofazimine 100 mg	Cfz	0.22	50	0.12	0.25	1
Cycloserine 250 mg	Cs	2.0	21	1.1	5.1	3
Delamanid 50 mg	Dlm	1.1	253	0.60	0.24	4
Ethambutol 400 mg	E	0.89	3.2	0.48	15	3
Ethionamide 250 mg	Eto	1.0	8.9	0.54	6.0	3
Imipenem/cilastatin 500 mg/500 mg	**Imp/Cls**	36	294	19	6.5	4
Isoniazid 300 mg	H	1.1	1.8	0.60	34	1–2.5
Kanamycin 1 g	**Km**	34	72	18	25	1
Levofloxacin 500 mg	Lfx	2.2	4.5	1.2	26	2
Linezolid 600 mg	Lzd	1.8	35	0.95	2.7	0.5–2
Moxifloxacin 400 mg	Mfx	2.0	16	1.1	6.7	1
PAS sodium salt 4 g	PAS	6.4	132	3.4	2.6	2
Pretomanid 200 mg	Pa	0.66	200	0.35	0.18	1
Prothionamide 250 mg	Pto	0.89	8.7	0.48	5.5	3
Pyrazinamide 400 mg	Z	1.0	2.1	0.53	26	4–5
Pyrazinamide 500 mg	Z	1.2	2.0	0.67	33	3–3.5
Rifampicin 300 mg	R	0.89	14	0.56	4.1	2
Rifapentine 150 mg	Rpt	0.58	22	0.31	1.4	8
Terizidone, 250 mg	Trd	0.97	175	0.52	0.3	3
**Fixed-dose combinations**						
HR 75 mg/150 mg	HR	1.0	4.3	0.53	12	4
HRZE 75 mg/150 mg/400 mg/275 mg	HRZE	1.2	8.8	0.65	7.4	4
**Fixed-dose combinations**						
Water for injection 5 ml	H2O	8.5	8.9	4.5	51	1

Drug abbreviation in **bold** = injectable antibiotic. *Data sources*: Own estimation based on previous micro-costing of medical supply import to the TB program [39], Global Drug Facility prices for TB drugs [56, 57], Médecins Sans Frontières and Ministry of Health Karakalpakstan TB guidelines [54], and World Health Organization TB guidelines [2, 5].

### 3.2 Number of tablets and injections per TB regimen

**Fig 1:**
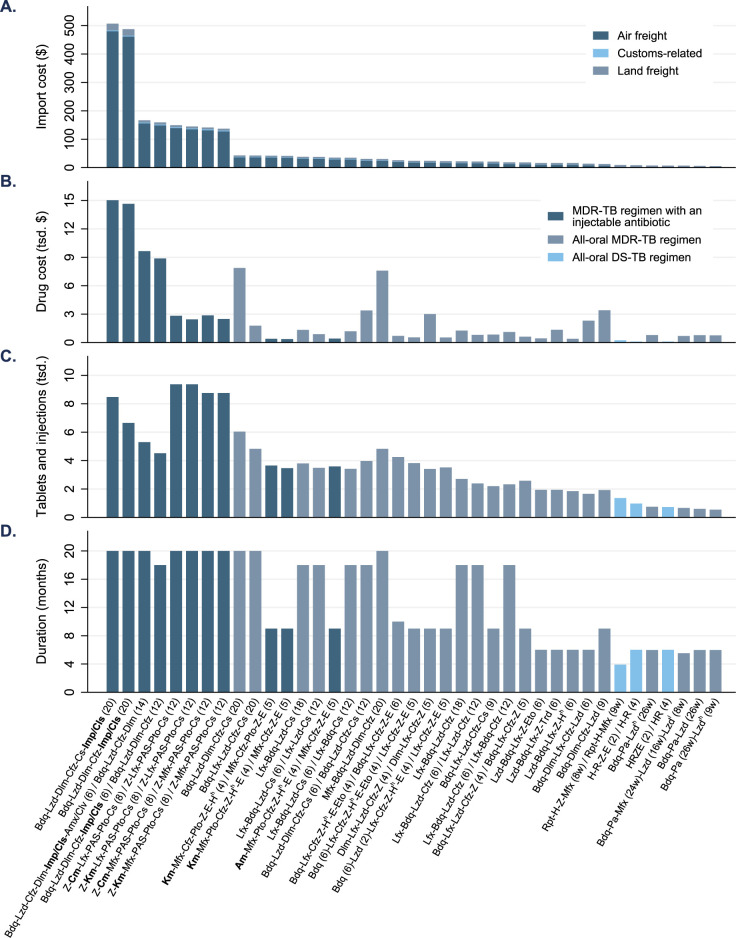
Import cost, drug cost, and drug amount of 39 longer, shorter, and short tuberculosis (TB) regimens: A prediction for a TB program in Karakalpakstan, Uzbekistan. **A.** Import cost per TB regimen. **B.** Drug cost per TB regimen. **C.** Number of tablets and injections per TB regimen. **D.** TB regimen duration. DS = drug-susceptible, MDR-TB = multidrug-resistant; drug abbreviation in **bold** = injectable antibiotic. TB regimens are sorted from highest to lowest import cost. Drug acronyms and formulations are described in **Table 1**.

The standard 6-month DS-TB regimen requires taking 730 tablets per treatment when fixed-dose combinations, which combine 2–4 antibiotics in one tablet, are used. When single-dose formulations instead of fixed-dose combination tablets are used for treatment, the number of tablets increases to 973 tablets for the standard DS-TB regimen. A new 4-month (17-week) DS-TB regimen requires 1358 tablets, mostly due to the need to take 8 tablets of rifapentine 150 mg daily to reach the recommended dose of 1200 mg per day (**Fig 1D** and **Table 2**).

**Table 2:** Duration, drug amount, drug cost, import weight and import cost of 39 longer, shorter, and short tuberculosis (TB) regimens for a TB program in Karakalpakstan, Uzbekistan.

TB drugs and duration of use (months or weeks [w])	Duration (months)	Tablets and injections per day	Tablets and injections	Drug cost ($)	Import weight (kg)	Import cost ($)	Import cost (% of drug cost)
**Drug-susceptible TB treatment**							
HRZE (2) / HR (4)	6	4	730	43	0.79	4.19	9.8
H-R-Z-E (2) / H-R (4)	6	3–10	973	64	0.94	5.31	8.2
Rpt-H-Z-Mfx (8w) / Rpt-H-Mfx (9w)	3.91	10–13	1358	233	1.1	6.07	2.6
**Multidrug-resistant TB treatment**							
***Longer regimens***							
Bdq-Lzd-Cfz-Dlm-**Imp/Cls**-Amx/Clv (6) / Bdq-Lzd-Cfz-Dlm (14)	20	6–16	5298	9653	31	167	1.7
Bdq-Lzd-Dlm-Cfz-Cs-**Imp/Cls** (20)	20	13–17	8474	15028	96	507	3.4
Bdq-Lzd-Dlm-Cfz-**Imp/Cls** (20)	20	10–14	6649	14644	92	487	3.3
Z-**Cm**-Lfx-PAS-Pto-Cs (8) / Z-Lfx-PAS-Pto-Cs (12)	20	15–16	9368	2827	28	149	5.3
Z-**Cm**-Mfx-PAS-Pto-Cs (8) / Z-Mfx-PAS-Pto-Cs (12)	20	14–15	8760	2869	27	141	4.9
Z-**Km**-Lfx-PAS-Pto-Cs (8) / Z-Lfx-PAS-Pto-Cs (12)	20	15–16	9368	2443	27	145	5.9
Z-**Km**-Mfx-PAS-Pto-Cs (8) / Z-Mfx-PAS-Pto-Cs (12)	20	14–15	8760	2485	26	137	5.5
Bdq-Lfx-Lzd-Cfz-Cs (20)	20	7–11	4824	1773	7.8	41	2.3
Bdq-Lzd-Dlm-Cfz-Cs (20)	20	9–13	6040	7874	7.8	42	0.53
Mfx-Bdq-Lzd-Dlm-Cfz (20)	20	7–11	4824	7588	5.3	29	0.38
Bdq-Lzd-Dlm-Cfz-**Imp/Cls** (6) / Bdq-Lzd-Dlm-Cfz (12)	18	6–14	4516	8877	30	159	1.8
Bdq-Lzd-Dlm-Cfz-Cs (6) / Bdq-Lzd-Cfz-Cs (12)	18	5–13	3969	3382	5.4	29	0.85
Lfx-Bdq-Lzd-Cfz (18)	18	4–8	2703	1257	3.7	20	1.6
Lfx-Bdq-Lzd-Cfz (6) / Lfx-Bdq-Cfz (12)	18	3–8	2326	1112	3.1	16	1.5
Lfx-Bdq-Lzd-Cfz (6) / Lfx-Lzd-Cfz (12)	18	4–8	2390	804	3.6	19	2.4
Lfx-Bdq-Lzd-Cs (18)	18	6–10	3798	1328	6.9	37	2.8
Lfx-Bdq-Lzd-Cs (6) / Lfx-Bdq-Cs (12)	18	5–10	3421	1184	6.2	33	2.8
Lfx-Bdq-Lzd-Cs (6) / Lfx-Lzd-Cs (12)	18	6–10	3485	876	6.8	36	4.1
***Shorter regimens***							
Bdq-Lfx-Cfz-Z-H^h^-E-Eto (4) / Bdq-Lfx-Cfz-Z-E (6)	10	11–20	4247	701	4.5	24	3.4
**Am**-Mfx-Pto-Cfz-Z-H^h^-E (4) / Mfx-Cfz-Z-E (5)	9	10–17	3589	415	6.3	33	8.1
**Km**-Mfx-Cfz-Pto-Z-E-H^h^ (4) / Mfx-Cfz-Pto-Z-E (5)	9	12–15	3650	394	7.6	41	10
**Km**-Mfx-Pto-Cfz-Z-H^h^-E (4) / Mfx-Cfz-Z-E (5)	9	10–16	3468	360	7.5	40	11
Bdq (6)-Lfx-Cfz-Z-H^h^-E-Eto (4) / Lfx-Cfz-Z-E (5)	9	11–20	3820	543	4.1	22	4.0
Bdq (6)-Lzd (2)-Lfx-Cfz-Z-H^h^-E (4) / Lfx-Cfz-Z-E (5)	9	11–18	3516	532	3.8	20	3.8
Bdq-Dlm-Cfz-Lzd (9)	9	6–10	1921	3406	1.9	10	0.29
Bdq-Lfx-Lzd-Cfz-Cs (9)	9	7–11	2195	833	3.5	19	2.2
Bdq-Lfx-Lzd-Cfz-Z (4) / Bdq-Lfx-Cfz-Z (5)	9	8–13	2578	618	3.0	16	2.5
Dlm-Lfx-Lzd-Cfz-Z (4) / Dlm-Lfx-Cfz-Z (5)	9	12–13	3407	3003	4.1	22	0.73
***Short regimens***							
Bdq-Dlm-Lfx-Cfz-Lzd (6)	6	8–12	1660	2308	2.1	11	0.48
Lzd-Bdq-Lfx-Z-Eto (6)	6	9.5–13.5	1934	432	2.6	14	3.2
Lzd-Bdq-Lfx-Z-H^h^ (6)	6	9–13	1843	391	2.5	13	3.4
Lzd-Bdq-Lfx-Z-Trd (6)	6	9.5–13.5	1934	1341	2.5	14	1.0
Bdq-Pa (26w)-Lzd^h^ (9w)	5.98	2–5	546	752	0.42	2.26	0.30
Bdq-Pa-Lzd (26w)	5.98	2–6	602	772	0.52	2.80	0.36
Bdq-Pa-Lzd^h^ (26w)	5.98	3–7	746	780	0.83	4.46	0.57
Bdq-Pa-Mfx (24w)-Lzd (16w)-Lzd^l^ (8w)	5.52	2.5–7	664	684	0.76	4.05	0.59

Drug abbreviation in **bold** = injectable antibiotic.

^h^ = high dose doubling the usual dose, e.g., 600 mg high-dose isoniazid (H^h^) per day

^l^ = low dose halving the usual dose, e.g., 300 mg low-dose linezolid (Lzd^l^) per day.

*TBTC Study 31

^†^formerly used in Karakapakstan

^††^presently used in Uzbekistan

^¶^BEAT TB trial

^§^NExT Study

^||^ZeNix TB trial

^#^Nix TB trial

**TB PRACTECAL trial. Drug acronyms and formulations are described in **Table 1**. *Data sources*: Own predictions for TB regimens formerly or presently recommended by the World Health Organization [42–44, 54, 55] and ClinicalTrials.gov registry [11–13, 17–19].

Conventional 20-month MDR-TB regimens, which included 8 months hospital-based treatment with the injectable antibiotics capreomycin or kanamycin in Karakalpakstan, require taking 8517–9125 tablets and 243 injections over the treatment course. Other longer 18–20-month MDR-TB regimens which include 6–20 months treatment with the injectable antibiotic imipenem/cilastatin involve taking 4151–7257 tablets and 365–1217 injections. Shorter 9–12-month MDR-TB regimens which include 4 months treatment with the injectable antibiotics amikacin or kanamycin involve taking 3346–3528 tablets and 122 injections.

All-oral TB regimens are without injectable antibiotics but can also require taking many tablets. In 18–20-month all-oral MDR-TB regimens, 2326–6040 tablets need to be taken. For 9–10-month all-oral MDR-TB regimens, 1921–4247 tablets should be taken over the treatment course. For short 24-weeks to 6-month MDR-TB regimens, the amount of TB drugs required for treatment decreases further to 546–1934 tablets.

### 3.3 Weight of TB regimens

Drugs for one course of DS-TB treatment were estimated to weigh 0.79–1.1 kg including drug packaging and transport packaging. Drugs for one course of longer MDR-TB treatment with injectables were estimated to weigh 26–96 kg as compared to 3.1–7.8 kg for longer all-oral MDR-TB regimens. One course of a shorter MDR-TB regimen with injectables was estimated to weigh 6.3–7.6 kg, one course of a shorter all-oral MDR-TB regimen 1.9–4.5 kg, and one course of a short MDR-TB regimen 0.42–2.6 kg. The exceptionally low weight of 0.42 kg of one 26-week MDR-TB regimen is due to a combination of 3 antibiotics only (bedaquiline, pretomanid, and linezolid) and their availability in formulations that require taking only 546 tablets over the short treatment course—fewer drugs than in any other of the assessed MDR-TB regimens (**Table 2**).

### 3.4 Costs and import costs of TB regimens

A standard 6-month DS-TB regimen with fixed-dose combinations was estimated to cost $43, to which import adds $4.19 (9.8% of the drug cost). If fixed-dose combinations are substituted by single-dose formulations, the costs per regimen were estimated to increase to $64 for drugs and $5.31 (8.2%) for import. The new 4-month (17-week) DS-TB regimen recommended by the WHO [2] was estimated to cost $233, to which import adds $6.07 (2.6%). For MDR-TB, the drug costs ($360–15,028) and import costs ($2.26–507 [0.29–11%]) of different regimens differ widely. Regimen costs and import costs were positively correlated with the regimen duration (Pearson’s *r* = 0.56 and *r* = 0.52, both P < 0.001) and the number of tablets and injections required by a regimen (*r* = 0.53 and *r* = 0.66, both P < 0.001) (**Fig 1**, **Table 2**, and **Table C in S1 File**).

Univariable linear regression analysis showed that the drug amount used in a regimen explains 27% of the variation in regimens’ drug cost, whereas regimen duration and whether a regimen contains injectables explain 30% and 20% of the variation in regimens’ drug cost, respectively. Regarding regimen import cost, the drug amount used in a regimen explains 42% of the import cost variation between regimens, whereas regimen duration and whether a regimen contains injectables explain 25% and 42% of the variation in the regimens’ import cost, respectively. In the multivariable regression model without interaction between independent variables, the drug cost of a regimen increases on average by $271 (95% CI: 109 to 434) per treatment month, but it was not significantly associated with the use of an injectable antibiotic. Regimen duration and use of an injectable drug were independent predictors of the regimen import cost and together explained 48% of the variation in import costs between regimens. The import cost of a TB regimen increases on average by $5.45 (95% CI: 1.65 to 9.26) per treatment month and by $133 (95% CI: 47 to 219) for regimens which include an injectable antibiotic (**Table 3**).

**Table 3:** Regression analysis of factors associated with the drug cost and import cost of a tuberculosis regimen.

Dependent variable and TB regimen characteristics	Univariable regressions	Multivariable regression	Multivariable regression with interaction
**Drug cost of a TB regimen ($)**			
Drugs per regimen (tsd.)	793 (196 to 1389)*		
Regimen duration (months)	344 (157 to 531)***	271 (109 to 434)**	
—All-oral regimens (month)^†^			172 (17 to 328)*
—Regimens with an injectable antibiotic (month)^†^			627 (243 to 1011)**
Regimen with injectable antibiotic	3861 (426 to 7295)*	2333 (-700 to 5366)	-4757 (-8631 to -883)*
Constant	Yes	-1445 (-2995 to 105)	-334 (-1597 to 929)
Adjusted R-squared	0.20 to 0.3	0.35	0.41
**Import cost of a TB regimen ($)**			
Drugs per regimen (tsd.)	30 (13 to 46)**		
Regimen duration (months)	10 (3.6 to 16)**	5.45 (1.65 to 9.26)**	
—All-oral regimens (month)^††^			1.72 (1.24 to 2.2)***
—Regimens with an injectable antibiotic (month)^††^			19 (7.64 to 30)**
Regimen with injectable antibiotic	164 (66 to 262)**	133 (47 to 219)**	-133 (-240 to -27)*
Constant	Yes	-43 (-85 to -0.54)*	-0.87 (-5.72 to 3.99)
Adjusted R-squared	0.25 to 0.42	0.48	0.60

N = 39. Coefficient (95% confidence interval).

*P < 0.05

**P < 0.01

***P < 0.001. Wald tests reject equality of coefficients: ^†^P = 0.032, ^††^P = 0.0038. Drugs per regimen and regimen duration were highly correlated and not included in the multivariable regressions together (Pearson’s *r* = 0.77, P < 0.001). Regression models including the drugs per regimen and of the import cost as a percentage of the drug cost are shown in the **Table D in S1 File**.

Multivariable regression with an interaction of regimen duration and all-oral treatment indicates that the procurement cost of TB regimens with and without an injectable antibiotic are affected differently by the regimen duration. Regimen duration, use of injectable drug, and their interaction were independent predictors of the regimen import cost and together explained 41% of the variation in the regimen cost and 60% of the variation in the cost to import a regimen. Other factors being equal, the drug cost of a regimen was estimated to increase on average by $172 (95% CI: 17 to 328) per treatment month for all-oral regimens and by $627 (95% CI: 243 to 1011) per treatment month for regimens with an injectable antibiotic; a Wald test rejects the equality of these coefficients (P = 0.032). Related to the higher weight of injectable TB drugs, the import cost of a regimen increases on average substantially more for regimens with an injectable antibiotic than for all-oral regimens ($19 [95% CI: 7.64 to 30] versus $1.72 [95% CI: 1.24 to 2.2] per treatment month; Wald test P < 0.0038). Shorter regimens with an injectable antibiotic can cost less to purchase and import than shorter all-oral regimens in the estimated regression model, but on average this relationship reverses as the treatment duration increases beyond a regimen duration of 10 (95% CI: 8.79 to 12) months for the drug cost and 7.79 (95% CI: 6.62 to 8.96) months for the import cost of a regimen.

Procuring medical supplies for TB drug administration (e.g., water for injection, syringes, or needles) can add to the drug regimen costs and import costs. To give an example, we estimated that importing 30.4167 units (0.26 kg) of 5 ml ampullae of sterile water, which are required for one month of daily injections of injectable antibiotics, adds $2.71 to a regimen’s monthly drug cost and $1.37 (51% of the cost of the sterile water) to a regimen’s monthly import cost (**Table 1**).

## 4 Discussion

### 4.1 Summary of findings

We used TB drug prices and import costs to estimate the cost of purchasing different DS-TB and MDR-TB regimens and importing them to a TB program in Karakalpakstan, Uzbekistan. The 39 assessed TB regimens require a substantially different amount of TB drugs (546 to 9368 tablets and injections) and thus have a different import weight (0.42 kg to 96 kg). Variations between drug regimens resulted from differences in the treatment duration as well as the combination of antibiotics that need to be taken daily—up to 20 tablets or 13–16 tablets plus 1–4 injection per day. For the shortest all-oral TB regimens assessed, we estimated import weights that are 2.5 to over 200 times smaller than those of the longest TB regimens with an injectable antibiotic. Therewith related, the estimated import cost for the assessed TB regimens to Karakalpakstan varied between $2.26 and $507. In a regression analysis, shorter TB treatment and all-oral TB treatment was associated with a lower import cost of the TB drugs required for a treatment course.

### 4.2 Comparison with previous findings

We are aware of one other study that accounted for TB programs’ international and national transportation costs as part of a cost-effectiveness analysis of TB control strategies in Africa and South East Asia [61]. In contrast to our bottom-up analysis of TB regimen import costs, the cost-effectiveness analysis derived a common import cost estimate for all imports based on trade-flow data for the entire country [30]. The costs of DS-TB and MDR-TB regimens without import costs, in turn, have been frequently calculated before, either as stand-alone cost analyses [42–44] or within cost and cost-effectiveness analyses of TB programs [45–48]. In a systematic review of DS-TB and MDR-TB treatment costs from 2015, a standard 6-month DS-TB regimen had a mean cost of $39 in lower-middle income countries, which is the World Bank income group to which Uzbekistan belongs to; a conventional longer regimen for MDR-TB had a mean cost of $2930 [47]. We estimated that the standard 6-month DS-TB regimen costs $43 at 2021 Global Drug Facility prices and that import to Karakalpakstan adds another $4.19. For conventional MDR-TB regimens with injectables, we estimated costs of $2443–2869 and additional import costs of $137–149.

The latest TB regimen costs reported by the MSF Access Campaign were based on 2020 Global Drug Facility prices and, like other studies, excluded import costs to a TB program. MSF reported costs of $905 for a 6-month MDR-TB regimen with a combination of bedaquiline, pretomanid, and linezolid (Global Drug Facility estimate). For other MDR-TB regimens with and without an injectable antibiotic costs between $461 and $10,389 were estimated with drug dosing for persons weighing 30–50 kg [44]. Using 2021 Global Drug Facility prices and drug dosing for persons weighing around 60 kg, we calculated costs of $780 (26 weeks) and $532–14,644 for these drug regimens and additional costs of import to Karakalpakstan of $4.46 and $9.95–487, respectively.

### 4.3 Practical implications

The decision on which TB regimens to provide has an impact on a TB program’s expenses for TB drug purchase and import. Using the regimen costs that were estimated in the study at hand, we compared the annual TB drug procurement costs of the TB program in Karakalpakstan under different treatment scenarios in a complementary modelling study [40]. The TB program in Karakalpakstan treated on average 2225 per year in 2016–20, of whom 30% were diagnosed with drug-resistant forms of TB. Transitioning from a 20-month all-oral to a 6-month all-oral MDR-TB regimen significantly reduced the annual MDR-TB drug import costs from $28 thousand to $3 thousand. In contrast, transitioning from a standard 6-month DS-TB regimen to a new and shorter 4-month DS-TB regimen significantly increased the program’s annual DS-TB drug import costs from $6.4 thousand to $9.3 thousand.

Import cost savings as a possible co-benefit of shorter regimens could allow a TB program to procure costlier drugs with a given budget. The presented findings suggest that shorter treatments tend to decrease import costs, especially when moving away from longer MDR-TB regimens that include injectable antibiotics. However, adapting the new 4-month DS-TB treatment with the currently available drug formulations was estimated to increase the cost of DS-TB treatment through higher drug costs and higher drug import costs. The higher import cost of the 4-month DS-TB regimen is mostly due to the current lack of fixed-dose combination tablets or rifapentine formulations that are optimized for the new 4-month DS-TB regimen. While high-dose tablets and fixed-dose combinations of TB drugs can help reduce drug import costs, both reduces the flexibility in how TB drugs can be used for different regimens or people.

The choice which TB regimens to provide with which type of drug formulations will likely be determined by several aspects, including, firstly, the clinical effectiveness, the frequency of adverse events, and the default rates, which differ between regimens [62]; secondly, context-specific factors, like national guidelines and locally approved and available drugs; and, thirdly, cost and cost-effectiveness considerations. Against this background, it is worthwhile to note that TB regimens which combine fewer antibiotics, require taking fewer drugs per day, and/or shorten treatment could help reduce import costs as well as the burden of taking a large drug amount for people on TB treatment (compare, e.g., [63–68]). Finding that the import cost of the drugs required for a TB regimen can vary widely, we recommend evaluating if the costs of importing require explicit consideration in cost and cost-effectiveness analyses and in financial planning of TB care. For a pragmatic import cost assessment, the overall magnitude of import costs could be assessed to gauge whether an in-depth analysis of the import cost of specific items might add information relevant to decision-making (compare [39]).

### 4.4 Strengths and limitations

This cost analysis has strengths and limitations. We used TB drug prices from a major global supplier and program-specific drug import costs to estimate the total cost of different TB regimens. Import costs included the transport costs for drugs and cargo packaging as well as customs-related charges. However, these import costs were assessed for one major shipment to the TB program in 2016 only and updated to 2021 prices. Unit weights were not available for some drugs and likely measured with low precision for other drugs. Both could have introduced error to the estimated drug-specific import costs. Regression results were based on a selected set of 39 regimens, limiting their external validity. Finally, some transport-related costs were not considered, such as costs of drug distribution from the central warehouse in Nukus to the point of care. Environmental costs of transport, disposal of item and cargo packaging, or other transport externalities, like health effects of transport emissions or noise, have also not been assessed. Therefore, from a societal perspective, the import costs of TB regimens can be higher than the presented estimates.

## 5 Conclusion

The amount of TB drugs transported and imported to a TB program can be large, as TB treatment requires taking combinations of antibiotics over extended periods of time. Hence, large amounts of drugs for treatment may not only pose a high pill burden on people treated for TB but can also cause sizable import costs to a TB program. Our findings suggest that drug import can cost $2.26–507 per TB regimen and add 0.29–11% to the TB drug cost for a TB program in Karakalpakstan. Shorter regimens, all-oral regimens, and optimized TB drug formulations can decrease the number and the weight of the drugs in a TB regimen and thus reduce the import cost for TB drugs. Related to a current unavailability of optimized drug formulations, a new 4-month DS-TB regimen had a higher import cost than the standard 6-month DS-TB regimen. For MDR-TB treatment, however, shorter and/or all-oral regimens tended to reduce the import cost per TB regimen.

## Supporting information

S1 File (DOCX)
