# Correction: The contribution of drug import to the cost of tuberculosis treatment: A cost analysis of longer, shorter, and short drug regimens for Karakalpakstan, Uzbekistan

**DOI:** 10.1371/journal.pgph.0003108

**Published:** 2024-04-02

**Authors:** Stefan Kohler, Norman Sitali, Jay Achar, Nicolas Paul

There are errors in the Funding and Competing Interests statements. The correct statements are:

**Funding:** Médecins Sans Frontières (MSF) provided support for the study in the form of salary for NS. The specific roles of this author are articulated in the ‘Authors’ section. MSF had no role in study design, data collection and analysis, decision to publish, or preparation of the manuscript.

**Competing Interests:** SK and NP consulted for Médecins Sans Frontières. NS works as a Medical Operations Manager for MSF, and thus received support from MSF in the form of salary. JA worked as TB medical advisor for the MSF Manson Unit. SK is an Academic Editor for *PLOS Global Public Health*. This does not alter our adherence to *PLOS Global Public Health* policies on sharing data and materials. There are no patents, products in development or marketed products associated with this research to declare.

There is an error with the “View Article” link for Reference 39. The correct link is: https://doi.org/10.34133/2021/9813732
