# Supplementary material for: The contribution of drug import to the cost of tuberculosis treatment: A cost analysis of longer, shorter, and short drug regimens for Karakalpakstan, Uzbekistan
Source: PLOS Glob Public Health. 2022 Aug 3;2(8):e0000567. doi: 10.1371/journal.pgph.0000567 (PMC10021759; doi:10.1371/journal.pgph.0000567)
Supplement: S1 File — (DOCX) [file pgph.0000567.s001.docx]

Table A. Characteristics of a shipment with tuberculosis (TB) drugs and other medicines to a TB program in Karakalpakstan, Uzbekistan.

| **Shipment characteristic** | **All medicines** | **TB drugs** | **Other medicines** |
| --- | --- | --- | --- |
| Items | 69 | 46 | 23 |
| Units (mil.) | 1.84 | 1.45 | 0.39 |
| Units per item (tsd.) | 4 (0.5 to 24) | 25 (11 to 62) | 1 (0.1 to 5.0) |
| Units per item line (tsd.) | 35 (8 to 595) | 35 (29 to 595) | 37 (4 to 587) |
| Unit price (ct) | 10 (2.6 to 58) | 10 (3.0 to 68) | 9.9 (1.6 to 58) |
| Unit weight (incl. item packaging; g) | 1.2 (0.7 to 15) | 1.0 (0.8 to 2.6) | 1.5 (0.7 to 40) |
| Order net weight (tons) | 7.2 | 3.5 | 3.7 |
| Order gross weight (incl. cargo packaging; tons) | 8.0 | 3.9 | 4.1 |
| Gross-to-net weight ratio | 1.11 | | |
| Order value (tsd. €) | 385 | 356 | 29 |
| Unit import cost (ct) | 0.5 (0.3 to 6.7) | 0.4 (0.3 to 1.1) | 0.7 (0.3 to 17) |
| Unit import cost (% of unit price) | 11 (4.2 to 27) | 5 (2.7 to 13) | 15 (6.8 to 49) |

Median (IQR). ct/€ = Euro at 2016 prices. ^*^Kruskal-Wallis tests reject an equality of the distribution across medicine groups for units per item (P < 0.001) and percentage unit import cost (P < 0.001), but not for units per item line (P = 0.72), unit price (P = 0.40), unit weight (P = 0.39), or unit import cost (P = 0.39). Order net weight and value were estimated by multiplying the unit weight and unit price, respectively, with the ordered quantity and summing-up over the items in a cargo. *Data source:* Own previous micro-costing of medical supply import to the TB program [1].

Table B. Dosing and missing import costs imputation of tuberculosis drugs.

| **TB drug and formulation** | **Dose per day** | **Daily units** | **Dosing source** | **Unit weight imputation^*^** | **Customs-related imputation^†^** |
| --- | --- | --- | --- | --- | --- |
| Amikacin 500 mg/2 ml | 1000 mg/  4 ml | 2 | WHO 2020 | No | Yes |
| Amoxicillin/clavulanic acid 500 mg/125 mg | 1000 mg/  250 mg | 2 |  | No | No |
| Bedaquiline 100 mg | 400 mg | 4 |  | 100 mg × median weight-per-dose of loose tablets 100–600 mg | No |
| Bedaquiline 100 mg | 200 mg | 0 or 2 |  |  |  |
| Bedaquiline 100 mg | 200 mg | 2 | ZeNix TB trial |  |  |
| Bedaquiline 100 mg | 100 mg | 1 |  |  |  |
| Capreomycin 1 g | 1000 mg | 1 | WHO 2019 | No | No |
| Clofazimine 100 mg | 100 mg | 1 | WHO 2020 | No | No |
| Cycloserine 250 mg | 750 mg | 3 |  | No | Yes |
| Delamanid 50 mg | 200 mg | 4 |  | No | No |
| Ethambutol 400 mg | 1200 mg | 3 |  | No | No |
| Ethionamide 250 mg | 750 mg | 3 |  | No | Yes |
| Imipenem/cilastatin 500 mg/500 mg | 4000 mg | 4 |  | 1 g × median weight-per-dose of capreomycin 1 g and kanamycin 1 g | No |
| Isoniazid 300 mg | 300 mg | 1 |  | No | No |
| Isoniazid 300 mg | 600 mg | 2 |  |  |  |
| Isoniazid 300 mg | 750 mg | 2.5 | NExT Study |  |  |
| Kanamycin 1 g | 1000 mg | 1 | WHO 2019 | No | No |
| Levofloxacin 500 mg | 1000 mg | 2 | WHO 2020 | 2 × weight-per-dose of levofloxacin 250 mg | Yes |
| Linezolid 600 mg | 300 mg | 0.5 | TB-PRACTECAL trial | No | Yes |
| Linezolid 600 mg | 600 mg | 1 | WHO 2020 |  |  |
| Linezolid 600 mg | 1200 mg | 2 |  |  |  |
| Moxifloxacin 400 mg | 400 mg | 1 |  | No | Yes |
| PAS sodium salt 4 g | 2 mg | 2 |  | 4 g × weight-per-dose of PAS sodium salt 9.2 g | Yes |
| Pretomanid 200 mg | 200 mg | 1 |  | 200 mg × median weight-per-dose of loose tablets 100–600 mg | Yes |
| Prothionamide 250 mg | 750 mg | 3 |  | No | Yes |
| Pyrazinamide 400 mg | 1600 mg | 4 |  | No | No |
| Pyrazinamide 400 mg | 2000 mg | 5 | MSF/MOH KK 2015 |  |  |
| Pyrazinamide 500 mg | 1500 mg | 3 | TBTC Study 31 | 500 g × weight-per-dose of pyrazinamide 400 mg | Yes |
| Pyrazinamide 500 mg | 1750 mg | 3.5 | NExT Study |  |  |
| Rifampicin 300 mg | 600 mg | 2 | WHO 2020 | No | No |
| Rifapentine 150 mg | 1200 mg | 8 | TBTC Study 31 | 150 mg × median weight-per-dose of tablets 100–600 mg in blister | Yes |
| Terizidone, 250 mg | 750 mg | 3 | WHO 2020 | 250 mg × median weight-per-dose of tablets 100–600 mg in blister | Yes |
| HR 75 mg/150 mg | 900 mg | 4 | WHO 2022 | No | No |
| HRZE 75 mg/150 mg/400 mg/275 mg | 3600 mg | 4 |  | No | Yes |
| Water for injection 5 ml | ≥5 ml | ≥1 | – | 5 ml × weight-per-dose of water for injection 10 ml | Yes |

HR = fixed-dose combination of isoniazid 75 mg/rifampicin 150 mg, HRZE = fixed-dose combination of isoniazid 75 mg/rifampicin 150 mg/pyrazinamide 400 mg/ethambutol 275 mg. ^*^Median of unit weights available in MSF Green List or unit weight of similar items was used to impute missing TB drug unit weights; air and land freight costs were calculated based on unit weights. ^†^Median tablet quantity imported in an item line in 2016 shipment was used to impute missing customs-related costs. *Dosing sources:* World Health Organization (WHO) TB guidelines 2019, 2020, and 2022 [2-4], Médecins Sans Frontières and Ministry of Health Karakalpakstan (MSF/MOH KK) TB guidelines 2015 [5], and ClinicalTrials.gov registry [6-11].

**Table C. TB regimen drug cost and import cost per person month and composition of import cost.**

| **TB drugs and duration of use (months or weeks [w])** | **Per person month ($)** | | | **Import cost composition ($)** | | |
| --- | --- | --- | --- | --- | --- | --- |
|  | **Total cost** | **Drug cost** | **Import cost** | **Air freight** | **Customs-related** | **Land freight** |
| **Drug-susceptible TB treatment** | | | | | | |
| HRZE (2) / HR (4) | 7.81 | 7.11 | 0.70 | 3.97 | 0.027 | 0.19 |
| H-R-Z-E (2) / H-R (4) | 12 | 11 | 0.88 | 4.72 | 0.36 | 0.23 |
| Rpt-H-Z-Mfx (8w) / Rpt-H-Mfx (9w) | 61 | 59 | 1.55 | 5.72 | 0.072 | 0.28 |
| **Multidrug-resistant TB treatment** | | | | | | |
| ***Longer regimens*** | | | | | | |
| Bdq-Lzd-Cfz-Dlm-**Imp/Cls**-Amx/Clv (6) / Bdq-Lzd-Cfz-Dlm (14) | 491 | 483 | 8.34 | 159 | 0.28 | 7.78 |
| Bdq-Lzd-Dlm-Cfz-Cs-**Imp/Cls** (20) | 777 | 751 | 25 | 483 | 0.45 | 24 |
| Bdq-Lzd-Dlm-Cfz-**Imp/Cls** (20) | 757 | 732 | 24 | 464 | 0.35 | 23 |
| Z-**Cm**-Lfx-PAS-Pto-Cs (8) / Z-Lfx-PAS-Pto-Cs (12) | 149 | 141 | 7.46 | 142 | 0.42 | 6.95 |
| Z-**Cm**-Mfx-PAS-Pto-Cs (8) / Z-Mfx-PAS-Pto-Cs (12) | 151 | 143 | 7.07 | 134 | 0.38 | 6.58 |
| Z-**Km**-Lfx-PAS-Pto-Cs (8) / Z-Lfx-PAS-Pto-Cs (12) | 129 | 122 | 7.25 | 138 | 0.67 | 6.74 |
| Z-**Km**-Mfx-PAS-Pto-Cs (8) / Z-Mfx-PAS-Pto-Cs (12) | 131 | 124 | 6.85 | 130 | 0.64 | 6.37 |
| Bdq-Lfx-Lzd-Cfz-Cs (20) | 91 | 89 | 2.07 | 39 | 0.26 | 1.92 |
| Bdq-Lzd-Dlm-Cfz-Cs (20) | 396 | 394 | 2.08 | 39 | 0.32 | 1.92 |
| Mfx-Bdq-Lzd-Dlm-Cfz (20) | 381 | 379 | 1.43 | 27 | 0.26 | 1.32 |
| Bdq-Lzd-Dlm-Cfz-**Imp/Cls** (6) / Bdq-Lzd-Dlm-Cfz (12) | 502 | 493 | 8.86 | 152 | 0.24 | 7.44 |
| Bdq-Lzd-Dlm-Cfz-Cs (6) / Bdq-Lzd-Cfz-Cs (12) | 189 | 188 | 1.59 | 27 | 0.21 | 1.33 |
| Lfx-Bdq-Lzd-Cfz (18) | 71 | 70 | 1.10 | 19 | 0.14 | 0.92 |
| Lfx-Bdq-Lzd-Cfz (6) / Lfx-Bdq-Cfz (12) | 63 | 62 | 0.90 | 15 | 0.12 | 0.75 |
| Lfx-Bdq-Lzd-Cfz (6) / Lfx-Lzd-Cfz (12) | 46 | 45 | 1.07 | 18 | 0.13 | 0.89 |
| Lfx-Bdq-Lzd-Cs (18) | 76 | 74 | 2.04 | 35 | 0.20 | 1.70 |
| Lfx-Bdq-Lzd-Cs (6) / Lfx-Bdq-Cs (12) | 68 | 66 | 1.84 | 31 | 0.18 | 1.54 |
| Lfx-Bdq-Lzd-Cs (6) / Lfx-Lzd-Cs (12) | 51 | 49 | 2.00 | 34 | 0.18 | 1.68 |
| ***Shorter regimens*** | | | | | | |
| Bdq-Lfx-Cfz-Z-H^h^-E-Eto (4) / Bdq-Lfx-Cfz-Z-E (6) | 73 | 70 | 2.40 | 23 | 0.19 | 1.11 |
| **Am**-Mfx-Pto-Cfz-Z-H^h^-E (4) / Mfx-Cfz-Z-E (5) | 50 | 46 | 3.72 | 32 | 0.16 | 1.56 |
| **Km**-Mfx-Cfz-Pto-Z-E-H^h^ (4) / Mfx-Cfz-Pto-Z-E (5) | 48 | 44 | 4.52 | 38 | 0.29 | 1.88 |
| **Km**-Mfx-Pto-Cfz-Z-H^h^-E (4) / Mfx-Cfz-Z-E (5) | 44 | 40 | 4.44 | 38 | 0.28 | 1.85 |
| Bdq (6)-Lfx-Cfz-Z-H^h^-E-Eto (4) / Lfx-Cfz-Z-E (5) | 63 | 60 | 2.42 | 21 | 0.17 | 1.01 |
| Bdq (6)-Lzd (2)-Lfx-Cfz-Z-H^h^-E (4) / Lfx-Cfz-Z-E (5) | 61 | 59 | 2.27 | 19 | 0.15 | 0.95 |
| Bdq-Dlm-Cfz-Lzd (9) | 380 | 378 | 1.11 | 9.39 | 0.10 | 0.46 |
| Bdq-Lfx-Lzd-Cfz-Cs (9) | 95 | 93 | 2.08 | 18 | 0.12 | 0.87 |
| Bdq-Lfx-Lzd-Cfz-Z (4) / Bdq-Lfx-Cfz-Z (5) | 70 | 69 | 1.75 | 15 | 0.10 | 0.73 |
| Dlm-Lfx-Lzd-Cfz-Z (4) / Dlm-Lfx-Cfz-Z (5) | 336 | 334 | 2.42 | 21 | 0.15 | 1.01 |
| ***Short regimens*** | | | | | | |
| Bdq-Dlm-Lfx-Cfz-Lzd (6) | 387 | 385 | 1.83 | 10 | 0.088 | 0.51 |
| Lzd-Bdq-Lfx-Z-Eto (6) | 74 | 72 | 2.27 | 13 | 0.10 | 0.63 |
| Lzd-Bdq-Lfx-Z-H^h^ (6) | 67 | 65 | 2.23 | 13 | 0.098 | 0.62 |
| Lzd-Bdq-Lfx-Z-Trd (6) | 226 | 224 | 2.25 | 13 | 0.10 | 0.63 |
| Bdq-Pa (26w)-Lzd^h^ (9w) | 126 | 126 | 0.38 | 2.13 | 0.029 | 0.10 |
| Bdq-Pa-Lzd (26w) | 129 | 129 | 0.47 | 2.63 | 0.032 | 0.13 |
| Bdq-Pa-Lzd^h^ (26w) | 131 | 130 | 0.74 | 4.21 | 0.040 | 0.21 |
| Bdq-Pa-Mfx (24w)-Lzd (16w)-Lzd^l^ (8w) | 125 | 124 | 0.73 | 3.83 | 0.035 | 0.19 |

Drug abbreviation in **bold** = injectable antibiotic. ^h^ = high dose doubling the usual dose, ^l^ = low dose halving the usual dose. Drug acronyms and formulations are described in **Table 1**. *Data sources:* Own estimation based on previous micro-costing of medical supply import to the TB program [1] and Global Drug Facility prices for TB drugs [12, 13].

**Table D. Regression analysis of factors associated with the drug cost and import cost of a tuberculosis regimen.**

|  | **Univariable regressions** | **Multivariable regression** | **Multivariable regression with interaction** | **Multivariable regression** | **Multivariable regression with interaction** |
| --- | --- | --- | --- | --- | --- |
| **Drug cost (% regimen cost)** |  |  |  |  |  |
| Drugs per regimen (tsd.) | 793 (196 to 1389)^*^ |  |  | 603 (-84 to 1290)+ |  |
| —All-oral regimen^§^ |  |  |  |  | 825 (207 to 1444)^*^ |
| —Regimen with injectable antibiotic^§^ |  |  |  |  | 393 (-800 to 1585) |
| Regimen duration (months) | 344 (157 to 531)^***^ | 271 (109 to 434)^**^ |  |  |  |
| —All-oral regimen^†^ |  |  | 172 (17 to 328)^*^ |  |  |
| —Regimen with injectable antibiotic^†^ |  |  | 627 (243 to 1011)^**^ |  |  |
| Regimen with injectable antibiotic | 3861 (426 to 7295)^*^ | 2333 (-700 to 5366) | -4757 (-8631 to -883)^*^ | 1480 (-2498 to 5458) | 3430 (-4587 to 11446) |
| Constant | Yes | -1445 (-2995 to 105)+ | -334 (-1597 to 929) | 33 (-1610 to 1677) | -542 (-1777 to 693) |
| Adjusted R-squared | 0.27 / 0.30 / 0.20 | 0.35 | 0.41 | 0.26 | 0.25 |
| **Import cost ($)** |  |  |  |  |  |
| Drugs per regimen (tsd.) | 30 (13 to 46)^**^ |  |  | 17 (1.84 to 32)^*^ |  |
| —All-oral regimen^\|\|^ |  |  |  |  | 7.53 (6.36 to 8.7)^***^ |
| —Regimen with injectable antibiotic^\|\|^ |  |  |  |  | 26 (-2.75 to 55) |
| Regimen duration (months) | 10 (3.6 to 16)^**^ | 5.45 (1.65 to 9.26)^**^ |  |  |  |
| —All-oral regimen^††^ |  |  | 1.72 (1.24 to 2.2)^***^ |  |  |
| —Regimen with injectable antibiotic^††^ |  |  | 19 (7.64 to 30)^**^ |  |  |
| Regimen with injectable antibiotic | 164 (66 to 262)^**^ | 133 (47 to 219)^**^ | -133 (-240 to -27)^*^ | 96 (7.94 to 185)^*^ | 12 (-144 to 168) |
| Constant | Yes | -43 (-85 to -0.54)^*^ | -0.87 (-5.72 to 3.99) | -26 (-66 to 14) | -1.12 (-2.98 to 0.75) |
| Adjusted R-squared | 0.42 / 0.25 / 0.42 | 0.48 | 0.60 | 0.48 | 0.49 |
| **Import cost (% regimen cost)** |  |  |  |  |  |
| Drugs per regimen (tsd.) | 0.23 (-0.06 to 0.52) |  |  | -0.37 (-0.88 to 0.14) |  |
| —All-oral regimen^#^ |  |  |  |  | -0.22 (-0.94 to 0.51) |
| —Regimen with injectable antibiotic^#^ |  |  |  |  | -0.51 (-1.2 to 0.2) |
| Regimen duration (months) | -0.037 (-0.18 to 0.11) | -0.17 (-0.31 to -0.024)^*^ |  |  |  |
| —All-oral regimen^¶^ |  |  | -0.07 (-0.21 to 0.076) |  |  |
| —Regimen with injectable antibiotic^¶^ |  |  | -0.52 (-0.7 to -0.34)^***^ |  |  |
| Regimen with injectable antibiotic | 3.2 (1.1 to 5.3)^**^ | 4.1 (2.2 to 6)^***^ | 11 (7.3 to 15)^***^ | 4.6 (1.4 to 7.9)^**^ | 6 (-0.61 to 12.6)+ |
| Constant | Yes | 4.2 (2 to 6.4)^***^ | 3.1 (0.8 to 5.5)^**^ | 3.3 (1.4 to 5.3)^**^ | 2.9 (0.35 to 5.5)^*^ |
| Adjusted R-squared | 0.014 / -0.021 / 0.23 | 0.32 | 0.44 | 0.26 | 0.25 |

N = 39. Coefficient (95% confidence interval). ^*^P < 0.05, ^**^P < 0.01, ^***^P < 0.001. Wald tests do not reject equality of coefficients: ^§^P = 0.52, ^||^P = 0.20, ^#^P < 0.56. Wald tests reject equality of coefficients: ^†^P = 0.032, ^††^P = 0.0038, ^¶^P < 0.001. Drugs per regimen and regimen duration were highly correlated and not included in the multivariable regressions together (Pearson's *r* = 0.77, P < 0.001).

**References**

1. Kohler, S., N. Sitali, and N. Paul, *A Framework for Assessing Import Costs of Medical Supplies and Results for a Tuberculosis Program in Karakalpakstan, Uzbekistan.* Health Data Science, 2021. **2021**: p. 1-13.

2. World Health Organization, *WHO consolidated guidelines on drug-resistant tuberculosis treatment*. 2019, Geneva: World Health Organization.

3. World Health Organization, *WHO operational handbook on tuberculosis. Module 4: treatment. Drug-resistant tuberculosis treatment*. 2020, Geneva: World Health Organization.

4. World Health Organization, *WHO consolidated guidelines on tuberculosis. Module 4: treatment. Drug-susceptible tuberculosis treatment.* 2022, Geneva: World Health Organization.

5. Médecins Sans Frontières and Ministry of Health of the Republic of Karakalpakstan, *Clinical guidelines on comprehensive TB treatment for drug-sensitive and drug-resistant tuberbulosis, Karakalpakstan, Uzbekistan*. 2015.

6. ClinicalTrials.gov. *Identifier: NCT02410772, TBTC Study 31: Rifapentine-containing tuberculosis treatment shortening regimens (S31/A5349)*. 2015 [cited 2022 June 26]; Available from: <https://clinicaltrials.gov/ct2/show/NCT02410772>.

7. ClinicalTrials.gov. *Identifier: NCT04062201, Building evidence for advancing new treatment for rifampicin resistant tuberculosis (RR-TB) comparing a short course of treatment (containing bedaquiline, delamanid and linezolid) with the current South African standard of care*. 2019 [cited 2022 June 26]; Available from: <https://clinicaltrials.gov/ct2/show/NCT04062201>.

8. ClinicalTrials.gov. *Identifier: NCT02454205, An open-label RCT to evaluate a new treatment regimen for patients with multi-drug resistant tuberculosis (NEXT)*. 2015 [cited 2022 June 26]; Available from: <https://clinicaltrials.gov/ct2/show/NCT02454205>.

9. ClinicalTrials.gov. *Identifier: NCT03086486, Safety and efficacy of various doses and treatment durations of linezolid plus bedaquiline and pretomanid in participants with pulmonary, XDR-TB, pre-XDR-TB or non-responsive/intolerant MDR-TB (ZeNix)*. 2017 [cited 2022 June 26]; Available from: <https://clinicaltrials.gov/ct2/show/NCT03086486>.

10. ClinicalTrials.gov. *Identifier: NCT02410772, A phase 3 study assessing the safety and efficacy of bedaquiline plus PA-824 plus linezolid in subjects with drug resistant pulmonary tuberculosis*. 2015 [cited 2022 June 26]; Available from: <https://clinicaltrials.gov/ct2/show/NCT02333799>.

11. ClinicalTrials.gov. *Identifier: NCT02589782, Pragmatic clinical trial for a more effective concise and less toxic MDR-TB treatment regimen(s) (TB-PRACTECAL)*. 2015 [cited 2022 June 26]; Available from: <https://clinicaltrials.gov/ct2/show/NCT02589782>.

12. Global Drug Facility, *Medicines Catalog October 2021*. 2021, Geneva: Stop TB Partnership/Global Drug Facility.

13. Global Drug Facility, *Medicines Catalog August 2018*. 2018, Geneva: Stop TB Partnership/Global Drug Facility.
